# A reproducible symptom-based computational phenotyping framework for longitudinal fibromyalgia analysis using real-world registry data: a cross-cohort validation study

**DOI:** 10.1186/s12911-026-03740-6

**Published:** 2026-08-05

**Authors:** Nazario Felix-Gonzalez, Jose-Maria Gomez-Arguelles, Ceferino Maestu-Unturbe

**Affiliations:** 1 Departamento de Arquitectura y Tecnología de Sistemas Informáticos, Escuela Técnica Superior de Ingenieros Informáticos, Universidad Politécnica de Madrid, Campus Montegancedo, Boadilla del Monte, Madrid, 28660 Spain; 2 Unidad de Fibromialgia y Dolor Crónico. Hospital Universitario San Rafael, Avenida Concha Espina, 32, Madrid, 28016 Spain; 3 Centro de Tecnología Biomédica, Universidad Politécnica de Madrid, Campus Montegancedo, Carretera M-40, km 38, Pozuelo de Alarcón, Madrid, 28223 Spain

**Keywords:** Medical informatics, Symptom-based computational phenotyping, Clinical symptom phenotypes, Unsupervised learning, External validation, Real-world data, Fibromyalgia

## Abstract

**Background:**

Fibromyalgia is a heterogeneous condition where clinical response to therapies like low-intensity repetitive Transcranial Magnetic Stimulation (Li-rTMS) varies significantly. Existing phenotyping is often limited by cross-sectional designs or lack of external validation. This study aims to develop and validate a reproducible informatics framework to identify stable symptom-based computational phenotypes and evaluate their longitudinal trajectories using multi-centre real-world data (RWD).

**Methods:**

We developed a dual-stage informatics pipeline for data curation and consistency filtering, which was applied to a registry of over 1,600 patients across three independent cohorts (Discovery, Structural Validation, and Longitudinal Validation). Unsupervised learning was performed using a K-means + + architecture on baseline clinical variables (FIQ, WPI, and SSS). Model generalizability was tested using a “frozen model” strategy, where centroids and scaling parameters from the discovery cohort were applied to the validation cohorts without re-fitting. Longitudinal stability was assessed by analysing transitions between these clinical symptom phenotypes across the treatment period.

**Results:**

The framework identified three distinct and stable symptom-based computational phenotypes: CP1 (Low Symptom Burden), CP2 (Pain-Dominant), and CP3 (High Multidimensional Burden). The curation pipeline ensured high data quality, maintaining structural consistency across all cohorts. CP1 demonstrated the highest longitudinal stability, acting as a clinical “sink,” while CP2 showed the most significant clinical improvement following Li-rTMS intervention. The results confirm that the identified symptom phenotypes are not artifacts of a specific dataset but represent reproducible clinical symptom states.

**Conclusions:**

The proposed informatics framework provides a reliable and transferable methodology for extracting stable clinical symptom phenotypes from heterogeneous real-world registries. By demonstrating cross-cohort validation and longitudinal consistency, this work offers a scalable template for personalized clinical decision-making and stratified management in chronic pain populations, aligning with FAIR data principles.

**Supplementary Information:**

The online version contains supplementary material available at https://doi.org/10.1186/s12911-026-03740-6.

## Background

Fibromyalgia is a chronic condition characterized by widespread pain and complex symptomatology, including fatigue, cognitive dysfunction, and sleep disturbances [1, 2]. While Low-intensity repetitive Transcranial Magnetic Stimulation (Li-rTMS) has emerged as a promising non-invasive neuromodulation therapy, clinical response remains highly heterogeneous among patients [3]. This variability poses a significant challenge for clinical decision-making, as practitioners lack reliable tools to predict individual therapeutic trajectories.

In recent years, the application of unsupervised learning to biomedical data has opened new avenues for understanding this clinical heterogeneity [4]. Clustering techniques have been used to identify patient “phenotypes” that may respond differently to interventions [5, 6]. However, much of the existing literature in fibromyalgia phenotyping is limited by its cross-sectional nature or reliance on single-center datasets, which often lack the longitudinal depth required to capture dynamic symptom evolution [7].

Furthermore, the transition from controlled clinical trials to Real-World Data (RWD) introduces significant informatics challenges, including clinical variability and heterogeneity inherent to multi-center registries [8]. There is a critical methodological gap regarding the stability and transferability of these phenotypes; a computational model developed in one clinical setting often may fail to generalize consistently to independent cohorts, undermining its utility for broader biomedical informatics applications [9, 10].

The significance of this work lies in addressing these gaps by moving from static observations to a structurally stable and externally validated framework. Following the FAIR principles for scientific data management [11], we implement a dual-stage cleaning protocol and a cross-cohort validation strategy to identify stable, transferable computational phenotypes. We present a pipeline for longitudinal phenotyping in fibromyalgia patients, with the primary objective of identifying multidimensional trajectories and evaluating their reproducibility across independent cohorts. Crucially, the resulting symptom-based computational phenotypes should be interpreted within the specific context of the clinical and diagnostic variables used to construct our baseline feature matrix, rather than as an exhaustive representation of the complete disease spectrum. By emphasizing transparent procedures and cross-cohort stability, this work provides a transferable computational framework for biomedical informatics research in chronic disease populations [12].

## Methods

This study implements a reproducible computational framework combining baseline phenotyping with longitudinal trajectory analysis under a fixed clustering architecture.

### Study design and setting

We conducted a retrospective multicentre study using real-world data (RWD) from a clinical registry of patients diagnosed with fibromyalgia according to the American College of Rheumatology (ACR) criteria. The study was designed to evaluate the reproducibility and generalizability of symptom-based computational phenotypes across independent cohorts.

The registry included longitudinal patient-reported outcomes collected across multiple clinical centres under standardized low-intensity repetitive transcranial magnetic stimulation (Li-rTMS) protocols. Data were stratified into three cohorts based on longitudinal completeness:

- **Cohort A (Discovery)**: complete three-stage follow-up (baseline, intermediate, end-of-treatment), used for phenotype derivation and longitudinal modelling.
- **Cohort B (External longitudinal validation)**: baseline and endpoint assessments, used for trajectory validation.
- **Cohort C (Structural validation)**: heterogeneous follow-up, used to assess phenotype transferability.

Model development and preprocessing were exclusively derived from the discovery cohort and subsequently applied to validation cohorts without re-fitting.

All data were pseudonymized and the study was approved by the corresponding ethics committee.

**Fig. 1:**
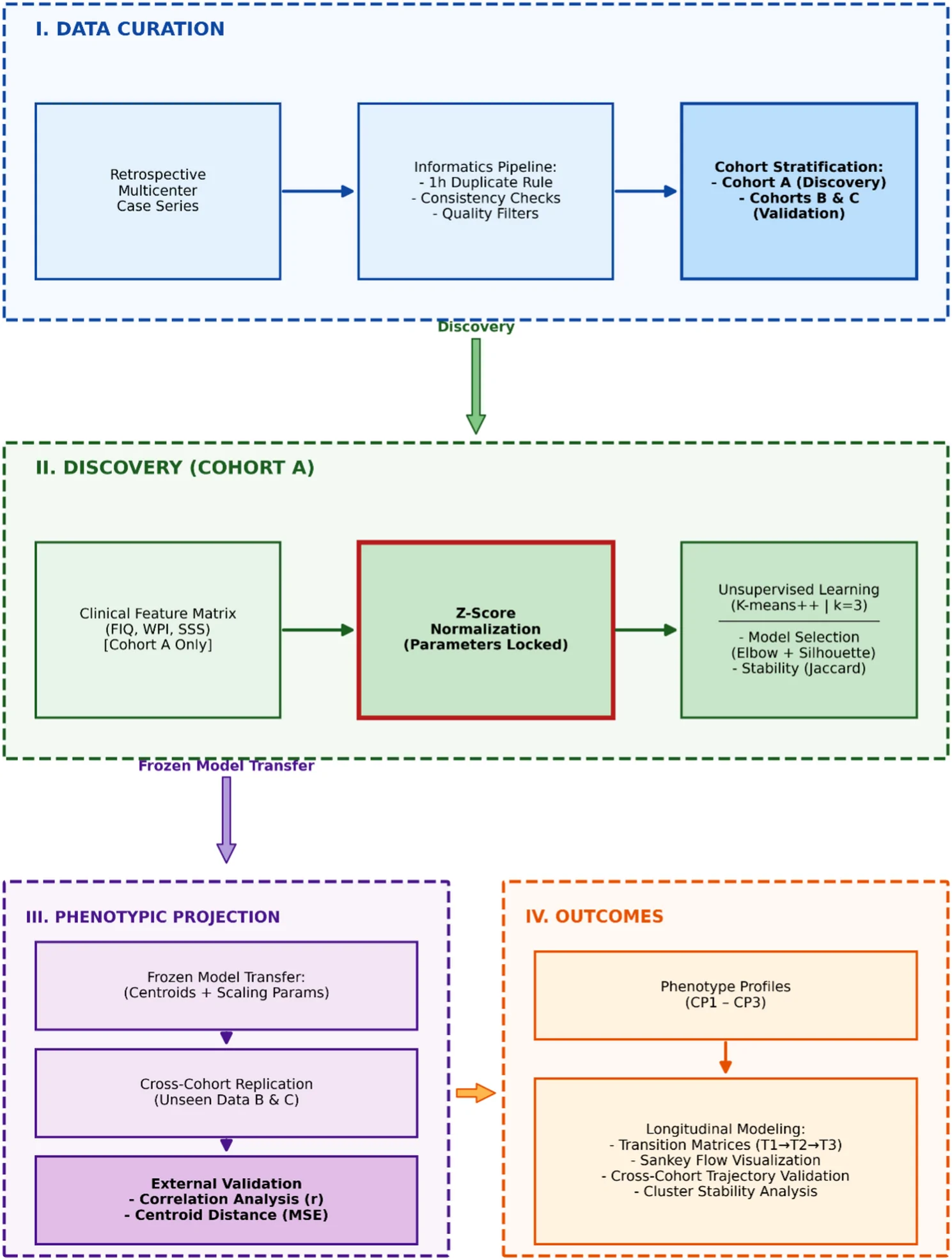
Symptom-based computational phenotyping framework for cross-cohort longitudinal analysis

Figure 1 provides an overview of the symptom-based computational phenotyping pipeline and its cross-cohort validation structure.

### Clinical feature matrix and participants

#### Inclusion criteria

Participants were included if they:

- were ≥ 18 years old.
- had a confirmed fibromyalgia diagnosis (ACR criteria).
- had complete baseline clinical assessments.

#### Feature matrix construction

Each patient was represented as a multidimensional vector derived from baseline measures:

- FIQ Total Score (0–100).
- WPI (0–19).
- SSS components: fatigue, sleep disturbance, cognitive symptoms (each 0–3).

No dimensionality reduction or feature selection was applied to preserve interpretability.

#### Clinical context

All participants received Li-rTMS under routine clinical practice conditions. The feature matrix was constructed exclusively from baseline data to ensure that phenotype derivation was independent of treatment effects.

#### Clinical protocol and longitudinal follow-up

All participants received Li-rTMS under standardized clinical practice conditions (frequency, intensity, and coil positioning). To ensure the consistency of the longitudinal trajectories, we verified the temporal adherence of the discovery cohort (Cohort A). Treatment intervals were analysed across the three predefined clinical phases: baseline (T1), intermediate (T2), and end-of-treatment (T3).

Mean temporal intervals were calculated for each clinical centre to assess potential inter-centre heterogeneity.

### Data curation and quality control

A sequential preprocessing pipeline was applied prior to analysis.

#### Technical de-duplication and temporal filtering

Records with identical participant and session identifiers within a 60-minute interval were considered duplicates; only the most recent entry was retained.

#### Missing data management

A complete-case approach was used. Records with missing values in core variables (FIQ, WPI, SSS) were excluded.

#### Demographic and longitudinal consistency

Only adult participants were included. Records with implausible demographic inconsistencies were removed.

#### Clinical consistency filtering

Two filters were applied:

- Plausibility rules: exclusion of contradictory clinical profiles.
- Low-variability detection: patients with zero variance in fatigue across all timepoints were excluded.

These steps ensured sufficient variability for clustering and longitudinal modelling.

Fatigue was selected as the reference dimension due to its central role as a core symptom of fibromyalgia and its contribution to multidimensional symptom profiles used in longitudinal and clustering studies.

#### Pipeline overview and analytical considerations

All preprocessing steps were applied in a predefined order prior to cohort stratification, ensuring reproducibility and separation from model development.

### Unsupervised learning architecture and model validation

#### Data preparation

Clustering was performed using baseline data from Cohort A. All features were standardized using *z-score* normalization, with parameters estimated from the discovery cohort and reused in validation datasets.

#### Clustering model

K-means + + was implemented (*random_state* = 42, *n_init* = 100) due to its robustness in low-dimensional continuous clinical spaces, centroid interpretability, computational reproducibility, and suitability for downstream centroid transfer across independent cohorts.

#### Number of clusters

The optimal number of clusters (*k* = 2–6) was evaluated using elbow and silhouette methods. A three-cluster solution was retained based on metric convergence and clinical interpretability.

#### Phenotype definition

Clusters were ordered by mean FIQ score to ensure consistent interpretation across analyses:

- CP1: low impact.
- CP2: moderate / pain-dominant.
- CP3: high multidimensional burden.

#### Stability and reproducibility

Cluster stability was assessed using 100 bootstrap iterations. Label alignment was performed using maximum Jaccard overlap. A mean *Jaccard index* > 0.75 was considered highly stable. The final model (centroids and scaling parameters) was serialized to enable exact reproducibility.

To further ensure the robustness of the three-cluster structure, a sensitivity analysis was conducted using the K-medoids algorithm with Gower’s distance, an approach specifically designed to handle mixed data types. This analysis allowed for the evaluation of the partition’s stability when employing a non-Euclidean distance metric. Technical reproducibility was further verified by re-executing the original K-means + + pipeline using the serialized parameters. The resulting consistency between the original and the re-executed models was quantified using the Adjusted Rand Index (ARI). These procedures were implemented to confirm that the proposed phenotypic framework is both computationally stable and resilient to variations in algorithmic assumptions.

#### External validation

The trained model was applied to Cohorts B and C using fixed scaling parameters and centroids. No model re-estimation was performed; this “frozen model” strategy ensures high computational efficiency, as the assignment of new observations requires only simple distance computations between the input feature vector and the predefined centroids. Given the low dimensionality of the feature space, this process enables low-latency phenotype assignment, facilitating near real-time deployment in clinical settings with minimal computational resource.

Phenotype transferability was evaluated by comparing centroid profiles across cohorts using:

- Pearson correlation.
- Mean squared error (MSE).

No model re-estimation was performed.

#### Clinical interpretability analysis

To validate the model’s decision-making process and ensure that the clustering architecture captures clinically meaningful patterns, we conducted a post-hoc interpretability analysis of the identified phenotypes. Given the centroid-based nature of the K-means algorithm, interpretability is inherently derived from the distribution of feature values within each cluster.

This analysis involved a comparative evaluation of baseline clinical variables—specifically the Fibromyalgia Impact Questionnaire (FIQ), WPI, and SSS components—across the three symptom-based computational phenotypes. By systematically mapping the model’s partitions against established gradients of fibromyalgia severity, we verified that phenotype assignments are consistently aligned with clinically recognized symptom structures. Importantly, this assessment is strictly limited to patient-reported symptom dimensions, validating the clinical utility and descriptive coherence of the partitions without assuming or implying underlying biological or biomarker-based distinction.

### Longitudinal analysis and phenotypic trajectories

#### Phenotype assignment over time

The fixed clustering model was applied to follow-up data. Observations were harmonized into three timepoints:

- T1: baseline.
- T2: intermediate.
- T3: end-of-treatment.

Phenotype assignment was performed without re-fitting.

#### Construction of individual trajectories

Individual trajectories were defined by phenotype sequences across T1–T3. Only patients with complete longitudinal data were included.

#### State transitions

Transition probabilities between phenotypes were computed for consecutive time intervals:

$$\:{P}_{ij}=\frac{{n}_{ij}}{\sum\nolimits_{k=1}^{K}{n}_{ik}}$$

where $\:{n}_{ij}$represents the number of patients transitioning from phenotype * i*at time * t*to phenotype * j*at time * t+1*, and * K*is the total number of phenotypes.

This formulation ensures row-normalized transition probabilities (i.e., ${\sum}_{j}{P}_{ij}=1$).

#### Phenotype consistency

Cluster stability over time was assessed by comparing feature means to baseline centroid confidence intervals (95%). This ensured that transitions reflected clinical change rather than model drift.

#### Longitudinal reproducibility

Trajectory patterns were compared between Cohorts A and B by evaluating phenotype-specific changes in FIQ and symptom severity, confirming consistency across independent datasets.

### Statistical analysis

Data normality was assessed using the *Shapiro–Wilk test*. Given non-normal distributions, non-parametric methods were applied:

- Between-group comparisons: *Kruskal–Wallis test* with *Dunn’s post-hoc* correction.
- Within-group longitudinal changes: *Wilcoxon signed-rank test*.

Cluster validity was assessed using silhouette scores, and robustness using the *Jaccard index*.

Statistical significance was set at *p* < 0.05.

All analyses were conducted in Python (v3.10) using SciPy and scikit-learn under fixed computational environments.

Throughout this manuscript, *n* denotes the number of unique patients, while *N* refers to the total number of clinical observations or records.

## Results

### Curation, cohort stratification, and protocol validation

#### Data curation pipeline impact

**Fig. 2:**
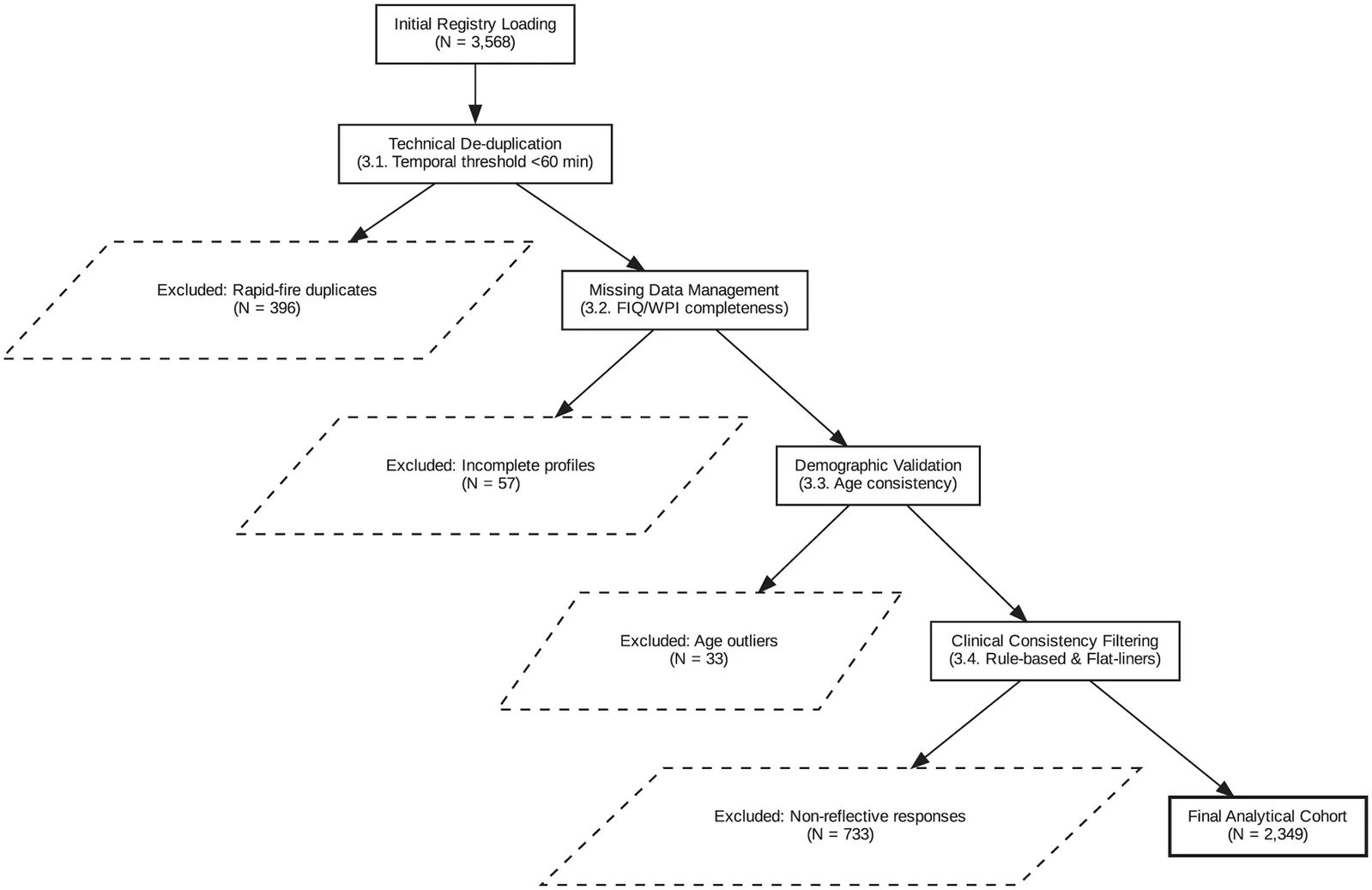
Informatics pipeline for data curation and quality control

The data curation pipeline transformed a heterogeneous multi-centre registry into a structured dataset suitable for computational phenotyping (Fig. 2).

A total of 3,568 (*N: Registers*) records were initially available. Sequential filtering steps resulted in progressive reduction:

- 396 records removed during de-duplication.
- 57 records excluded due to missing clinical variables.
- 33 records excluded for demographic inconsistencies.
- 733 records removed during clinical consistency filtering.

The final analytical dataset comprised 2,349 observations, reflecting a balance between data quality and retention of clinically meaningful variability.

The largest reduction occurred during clinical consistency filtering, indicating that a substantial proportion of raw registry data contained non-informative or inconsistent patterns.

#### Cohort stratification

The curated dataset was partitioned into three cohorts based on longitudinal data availability:

- Cohort A (*n* = 268): complete three-point follow-up (discovery).
- Cohort B (*n* = 97): baseline and endpoint (external longitudinal validation).
- Cohort C (*n* = 916): heterogeneous follow-up (structural validation).

This stratification enabled strict separation between phenotype discovery, validation, and generalisation (Table 1).

**Table 1:** Summary of study cohorts and longitudinal data characteristics

Cohort	Longitudinal Data Availability	Patients (*n*)	Observations (*N*)	Analytical Purpose
Cohort A (Discovery)	Three-point longitudinal follow-up	268	849	Discovery cohort for symptom-based computational phenotyping
Cohort B (External Validation)	Baseline and endpoint assessments	97	215	External longitudinal validation of trajectories
Cohort C (Structural Validation)	Heterogeneous longitudinal follow-up	916	1285	Cross-cohort structural validation of phenotypes

Notes: The table defines the stratified groups used for the cross-cohort validation study. Cohort A represents the discovery set used to define the core symptom-based computational phenotypes. Cohort B serves for external validation of the longitudinal patterns (trajectories) identified in the discovery phase. Cohort C comprises a large, heterogeneous multi-center sample used to ensure the structural stability and generalizability of the phenotypes across the real-world dataset

To ensure the robustness of the longitudinal findings, we assessed the temporal adherence of the Cohort A (*n* = 268) to the standard Li-rTMS protocol. Clinical follow-up was structured into three phases: baseline (T1), intermediate (T2), and final (T3). Descriptive statistics regarding the temporal intervals between phases (T1–T2, T2–T3, and T1–T3) across clinical sites are summarized in Supplementary Table ST1. To maintain data privacy and centre anonymity, results are presented as Centre 1 (high-volume), Centre 2 (high-volume), and ‘Others’ (grouping smaller clinical sites). Although minor inter-centre variations in treatment scheduling were observed—reflective of real-world clinical practice—with significant differences identified between the two high-volume centres across all intervals (p < 0.01), these deviations were not statistically associated with the phenotypic transition probabilities. This confirms the stability and reproducibility of the clustering architecture across the cohort, despite the observed scheduling heterogeneity.

### Baseline symptom-based computational phenotyping

#### Cluster identification

Unsupervised clustering identified three distinct symptom-based computational phenotypes.

**Fig. 3:**
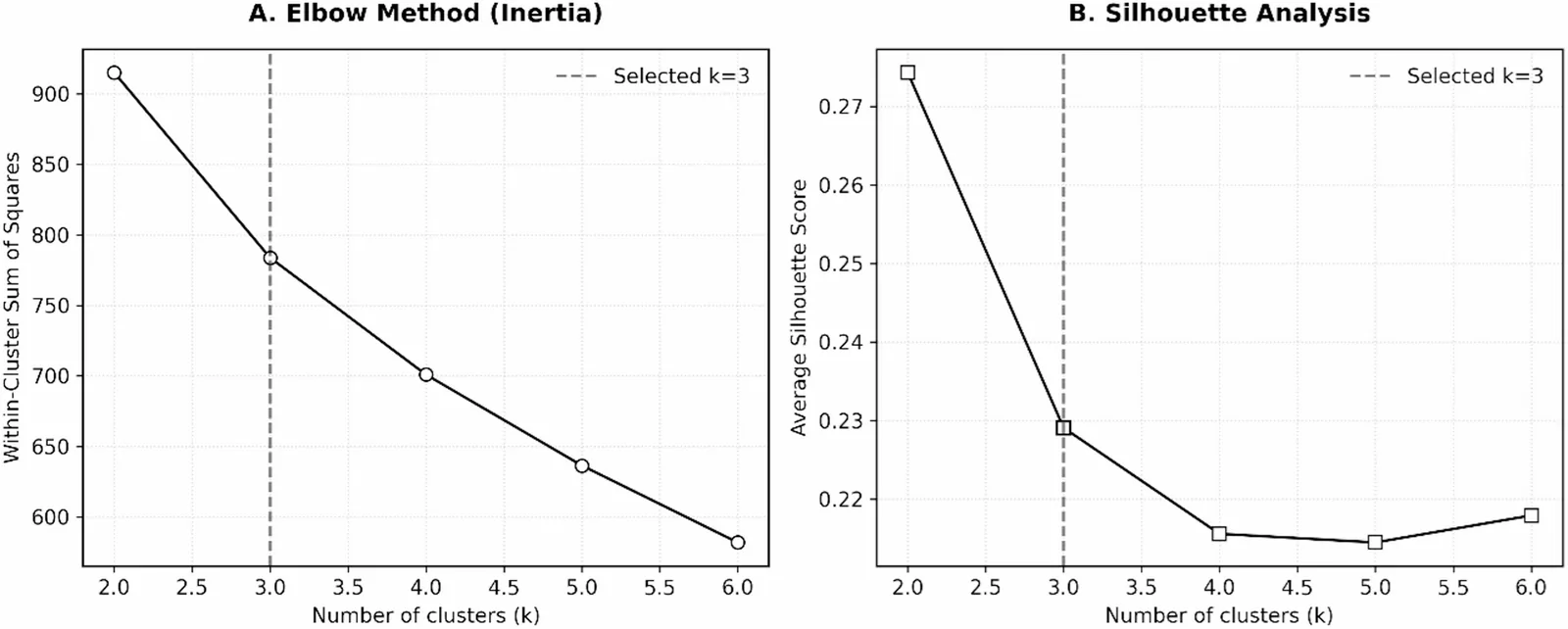
Cluster validation analysis. (**A**) Elbow method showing WCSS reduction; (**B**) Silhouette score analysis for optimal k selection

Model selection metrics supported the three-cluster solution (Fig. 3):

- *Elbow* analysis showed an inflection point at *k* = 3.
- *Silhouette* scores indicated adequate cluster separation.

#### Clinical characterization

The three phenotypes exhibited clearly differentiated clinical profiles (Table 2).

- **CP1 (Low Impact)**: consistently low symptom burden.
- **CP2 (Pain-Dominant)**: high pain with intermediate symptom severity.
- **CP3 (High Impact)**: elevated values across all dimensions.

No differences were observed in age or sex (*p* > 0.2), confirming that clustering was driven by clinical rather than demographic variables.

In contrast, all clinical variables differed significantly (*p* < 0.001), demonstrating strong discriminative capacity.

**Table 2:** Baseline demographic and clinical characteristics of patients across the three identified symptom-based computational phenotypes

Variable	CP1: Low Impact (*n* = 69)	CP2: Moderate (*n* = 104)	CP3: High Impact (*n* = 95)	*p*-value
Age (years)	50.40 ± 11.20	51.80 ± 10.50	51.30 ± 10.90	0.245
Female participants n (%)	63 (91.3%)	96 (92.3%)	87 (91.6%)	0.892
FIQ Total Score (0-100)	57.71 ± 11.72	72.82 ± 8.33	79.38 ± 8.69	< 0.001
WPI Score (0–19)	7.93 ± 3.98	14.34 ± 3.36	14.42 ± 3.97	< 0.001
Fatigue (SSS; 0–3)	1.94 ± 0.54	1.92 ± 0.27	2.99 ± 0.10	< 0.001
Sleep Disturbance (SSS; 0–3)	1.58 ± 0.74	2.22 ± 0.71	2.65 ± 0.60	< 0.001
Cognitive Symptoms (SSS;0–3)	0.99 ± 0.74	1.82 ± 0.66	2.13 ± 0.78	< 0.001

**Fig. 4:**
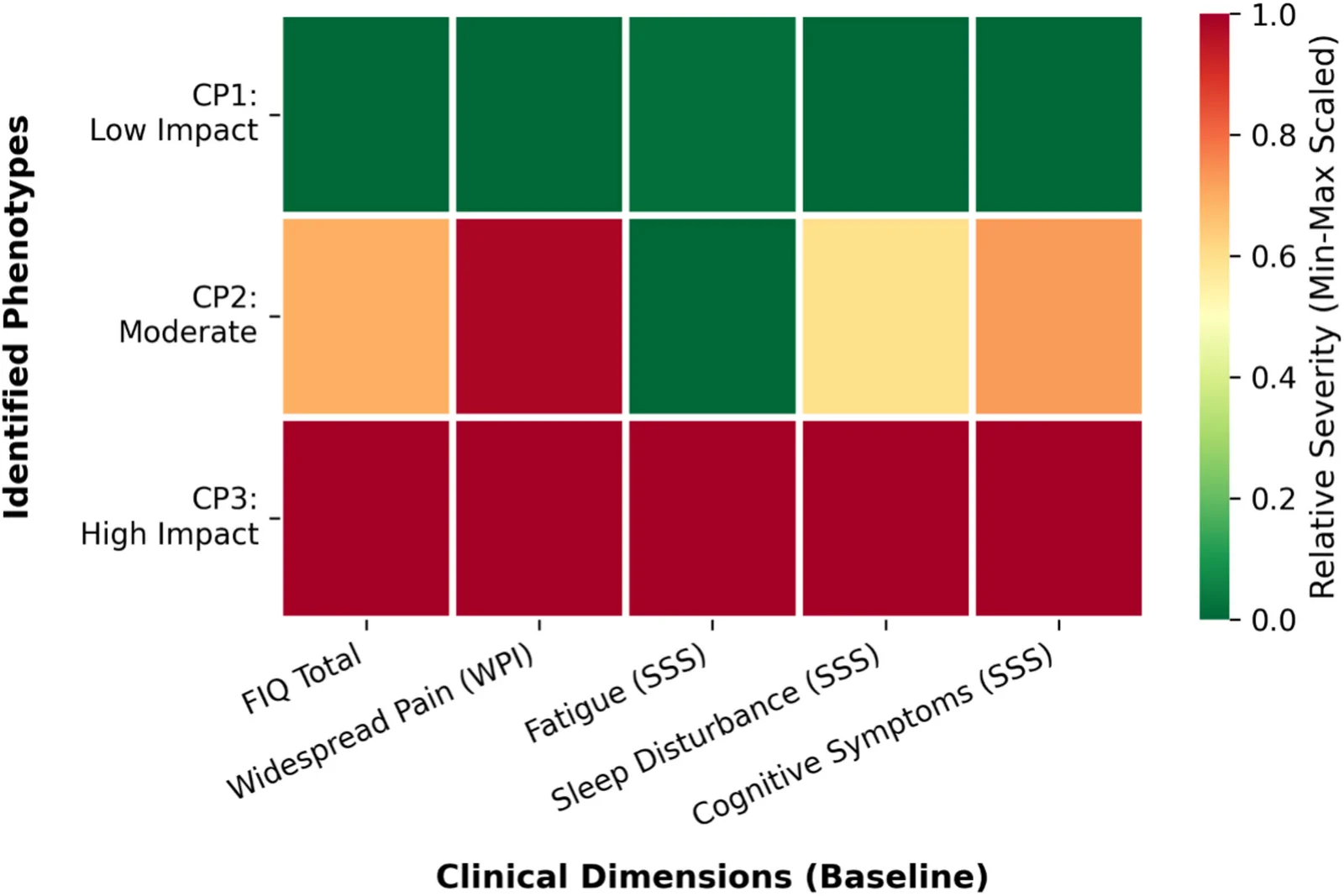
Multidimensional clinical profiles of symptom-based computational phenotypes. Clinical variables were normalized to allow cross-dimensional comparison. Each row represents a phenotype (CP1–CP3) and each column a clinical variable. The heatmap illustrates a structured gradient of symptom burden, with CP1 showing consistently low values, CP3 showing uniformly high severity, and CP2 presenting a distinct pain-dominant profile. This visualization supports the internal coherence and discriminative structure of the clustering solution

These differences are further illustrated in Fig. 4, which provides a visual representation of the multidimensional structure of the phenotypes.

The heatmap highlights a clear gradient of symptom severity from CP1 to CP3, supporting the internal consistency of the clustering solution. In addition, CP2 exhibits a distinct pattern characterized by disproportionately elevated pain (WPI) relative to other symptom domains, reinforcing its identification as a pain-dominant phenotype.

#### Interpretability analysis and model validation

To validate the model’s internal decision-making process, we performed a post-hoc interpretability analysis of the clinical features driving phenotype assignment. Given the centroid-based nature of the K-means clustering algorithm, interpretability arises directly from the feature-wise distribution of clinical variables across clusters.

The consistent symptom severity gradient observed across all clinical domains (Table 2), together with the distinct multidimensional patterns identified in the heatmap (Fig. 4), confirms that the clustering structure aligns with established clinical markers of fibromyalgia. In particular, the progressive transition from CP1 (low impact) to CP3 (high multidimensional burden) reflects a coherent and clinically meaningful increase in symptom severity across FIQ, WPI, and SSS components.

These findings demonstrate that the clustering solution is not driven by arbitrary data artifacts, but instead captures structured and clinically coherent variations in patient profiles. This post-hoc validation supports both the interpretability and the clinical reliability of the proposed symptom-based computational phenotyping framework.

#### Cluster stability

*Bootstrap analysis* (100 iterations) confirmed the robustness of the clustering solution. The *mean Jaccard index* across all clusters was 0.776 ± 0.14. Specifically, CP3 showed the highest stability (*mean Jaccard* = 0.826 ± 0.10), while CP1 showed greater variability (*mean Jaccard* = 0.707 ± 0.18), reflecting higher internal heterogeneity. All clusters exceeded the accepted stability threshold (> 0.60/0.70), indicating a reproducible phenotype structure.

**Fig. 5:**
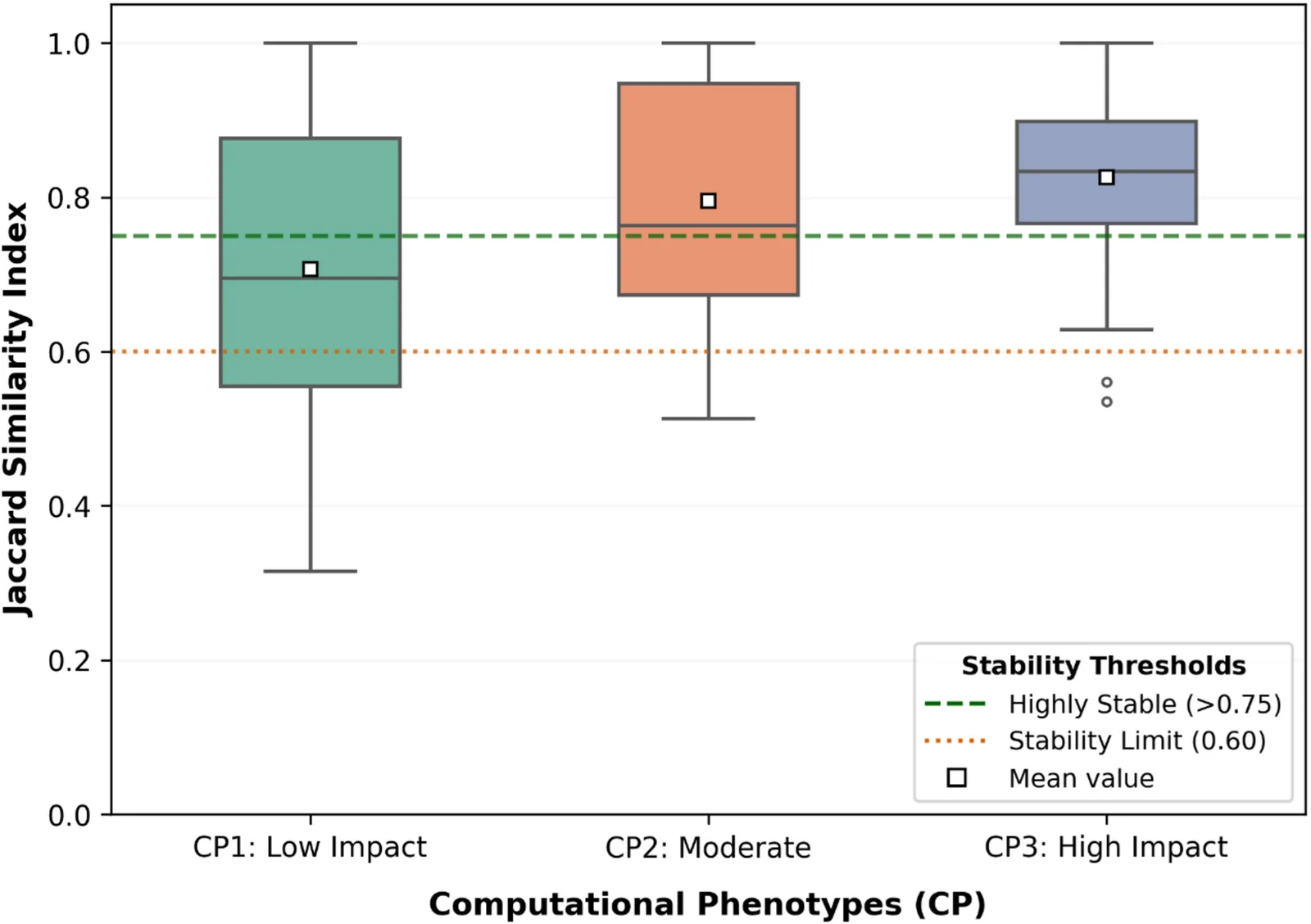
Cluster stability based on bootstrap resampling (*n* = 100). Stability was assessed using the Jaccard similarity index across symptom-based computational phenotypes. Boxplots represent the distribution of stability values, with dashed lines indicating reference thresholds for moderate (0.60) and high stability (0.75). CP3 shows consistently high stability with low dispersion, whereas CP1 exhibits greater variability, reflecting increased heterogeneity. Overall, all phenotypes remain within acceptable stability ranges, supporting the robustness of the clustering solution

The distribution of stability values (Fig. 5) further illustrates the robustness of the clustering solution. CP3 shows consistently high stability with limited dispersion, indicating a well-defined and compact phenotype. In contrast, CP1 exhibits greater variability, suggesting increased heterogeneity within lower-symptom profiles. Despite this variability, all phenotypes remain above accepted stability thresholds, supporting the reliability of the overall clustering structure.

Furthermore, to assess the sensitivity of the identified phenotypic structure to different distance metrics and clustering assumptions, we performed a secondary analysis using the K-medoids algorithm with Gower’s distance.

The comparison between the original K-means + + model and the K-medoids-based partition (Supplementary Figure SF1) yielded an Adjusted Rand Index (ARI) of 0.19, indicating modest agreement at the level of individual patient assignments.

Inspection of the cross-tabulation revealed that agreement was highest for the extreme phenotypes (CP1 and CP3), whereas the intermediate phenotype (CP2) showed substantial dispersion across adjacent clusters. This pattern suggests that patients located near phenotype boundaries are particularly sensitive to the choice of distance metric and clustering algorithm, reflecting the continuous nature of fibromyalgia symptom severity rather than the existence of sharply separated clinical categories.

Importantly, despite differences in individual assignments, clinical profiling of the resulting partitions demonstrated that both methodologies consistently recovered a comparable severity gradient, preserving the hierarchical ordering of symptom burden across phenotypes (CP1 < CP2 < CP3). Thus, the underlying clinical archetypes remained stable across clustering approaches.

Additionally, re-execution of the original K-means + + pipeline using the serialized model parameters demonstrated high technical reproducibility (ARI = 0.76), supporting the internal stability of the proposed framework under fixed methodological conditions.

Taken together, these findings suggest that while exact cluster membership may vary across algorithms, particularly for intermediate cases, the overall clinical structure and severity continuum identified by the three-phenotype solution remain consistent across methodological assumptions.

### Cross-cohort structural validation

Clinical profiles associated with each phenotype were preserved across independent cohorts (Table 3).

**Table 3:** Cross-cohort comparison of clinical profiles across symptom-based computational phenotypes

Variable	CP1			CP2			CP3		
	(A)	(B)	(C)	(A)	(B)	(C)	(A)	(B)	(C)
FIQ Total Score	57.7 ± 11.7	61.7 ± 9.1	57.1 ± 12.7	72.8 ± 8.3	72.6 ± 7.8	74.0 ± 8.1	79.4 ± 8.7	76.5 ± 10.3	80.4 ± 8.9
WPI Total	7.9 ± 4.0	8.1 ± 5.2	7.5 ± 4.3	14.3 ± 3.4	13.2 ± 4.0	14.0 ± 3.5	14.4 ± 4.0	14.6 ± 4.3	14.3 ± 4.0
Fatigue (SSS)	1.94 ± 0.54	1.67 ± 0.62	1.78 ± 0.76	1.92 ± 0.27	1.97 ± 0.18	1.94 ± 0.31	2.99 ± 0.10	3.00 ± 0.00	2.99 ± 0.12
Sleep (SSS)	1.58 ± 0.74	1.93 ± 0.59	1.65 ± 0.91	2.22 ± 0.71	2.34 ± 0.70	2.24 ± 0.64	2.65 ± 0.60	2.76 ± 0.43	2.62 ± 0.56
Cognitive (SSS)	0.99 ± 0.74	1.00 ± 0.76	0.95 ± 0.75	1.82 ± 0.66	1.88 ± 0.71	1.82 ± 0.70	2.13 ± 0.78	2.42 ± 0.64	2.21 ± 0.75

Notes: Values are presented as mean ± standard deviation. A: Discovery cohort; B: external validation cohort; C: structural validation cohort

Mean values for FIQ, WPI, and SSS components showed minimal variation between cohorts A, B, and C.

- CP1 maintained low symptom burden.
- CP2 retained a pain-dominant profile.
- CP3 remained consistently high across all dimensions.

These findings demonstrate that the phenotype definitions derived in the discovery cohort are transferable to independent populations.

### Longitudinal phenotype dynamics

#### Symptom trajectories

All phenotypes exhibited improvement over time (Table 4).

- CP2 showed the greatest reduction in FIQ (− 28.2%).
- CP1 also improved substantially (− 11.6%).
- CP3 showed more moderate but consistent improvement (− 27%).

All within-group changes were statistically significant (*p* < 0.05).

**Table 4:** Longitudinal changes in clinical measures in the discovery cohort (Cohort A)

Variable	Assessment	CP1 (*n* = 69)	CP2 (*n* = 104)	CP3 (*n* = 95)
FIQ Total Score	Baseline	46.61	69.87	75.93
	Final	41.19	50.14	55.45
	Relative Change	-11.63%	-28.24%	-26.97%
Fatigue (0–3)	Baseline	1.54	1.9	2.97
	Final	1.36	1.63	2.08
	Relative Change	-11.69%	-14.21%	-29.97%
WPI Total	Baseline	7.47	13.91	11.84
	Final	6.76	9.07	10.37
	Relative Change	-9.50%	-34.79%	-12.42%
Sleep SSS (0–3)	Baseline	1.33	2.28	2.5
	Final	1.29	1.74	2.24
	Relative Change	-3.01%	-23.68%	-10.40%

Notes: Values represent mean scores. Relative change is calculated as the percentage difference between baseline and final follow-up. All within-group longitudinal changes were statistically significant (Wilcoxon signed-rank test, *p* < 0.05)

#### Transition patterns

**Fig. 6:**
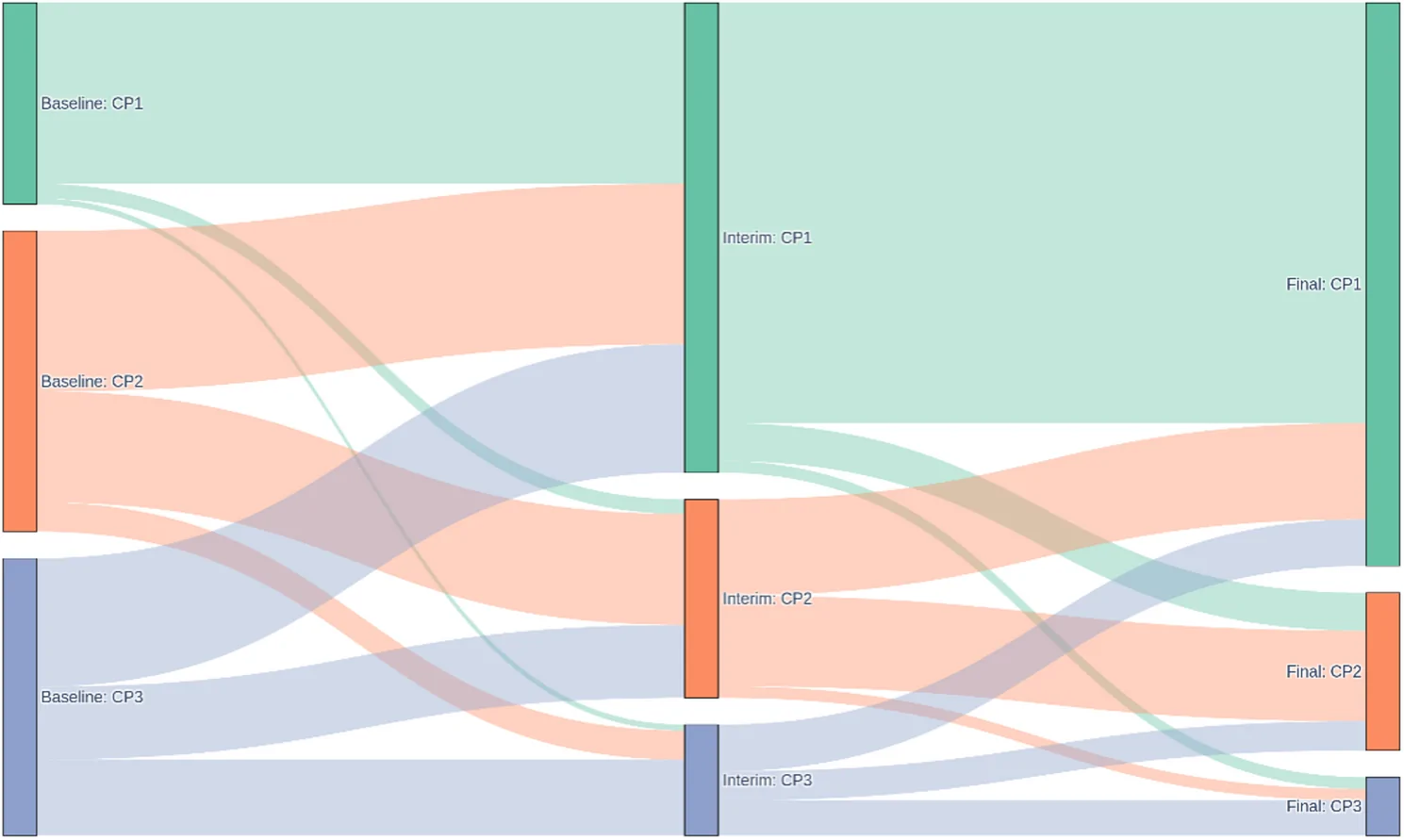
Multi-stage Sankey diagram illustrating phenotype transitions from baseline (T1) to intermediate (T2) and final follow-up (T3). Nodes represent symptom-based computational phenotypes at each timepoint (CP1–CP3), and link widths correspond to the number of patients transitioning between states

Phenotypic transitions revealed distinct dynamic behaviours (Table 5; Fig. 6):

- CP1 (Low Impact) high stability: This phenotype functioned as a primary clinical “sink,” with approximately 89.9% (T1→T2) and 89.4% (T2→T3) of patients remaining stable in this group.
- CP2 (Moderate) high mobility: This cluster showed the most significant early response, with 53.4% of patients transitioning to the milder CP1 in the first interval and 48.5% in the final stage.
- CP3 (High Impact) heterogeneous trajectories: Patients in this group exhibited high mobility toward improvement, with 46.3% (T1→T2) and 42.1% (T2→T3) successfully migrating to CP1, while persistence in the severe state was limited to 27.4% and 31.6% respectively.

These results indicate that the identified phenotypes capture not only baseline differences but also distinct, predictable patterns of clinical evolution and recovery under treatment.

**Table 5:** Longitudinal transition probabilities (%) between symptom-based computational phenotypes across treatment phases

Baseline Phenotype	Transition Interval	CP1 (Low Impact)	CP2 (Moderate)	CP3 (High Impact)
CP1 (Low)	T1 → T2	89.86%	7.25%	2.89%
	T2 → T3	89.44%	8.07%	2.49%
CP2 (Moderate)	T1 → T2	53.40%	36.89%	9.71%
	T2 → T3	48.53%	45.59%	5.88%
CP3 (High)	T1 → T2	46.32%	26.32%	27.36%
	T2 → T3	42.11%	26.32%	31.57%

### External longitudinal validation

#### Trajectory reproducibility

**Fig. 7:**
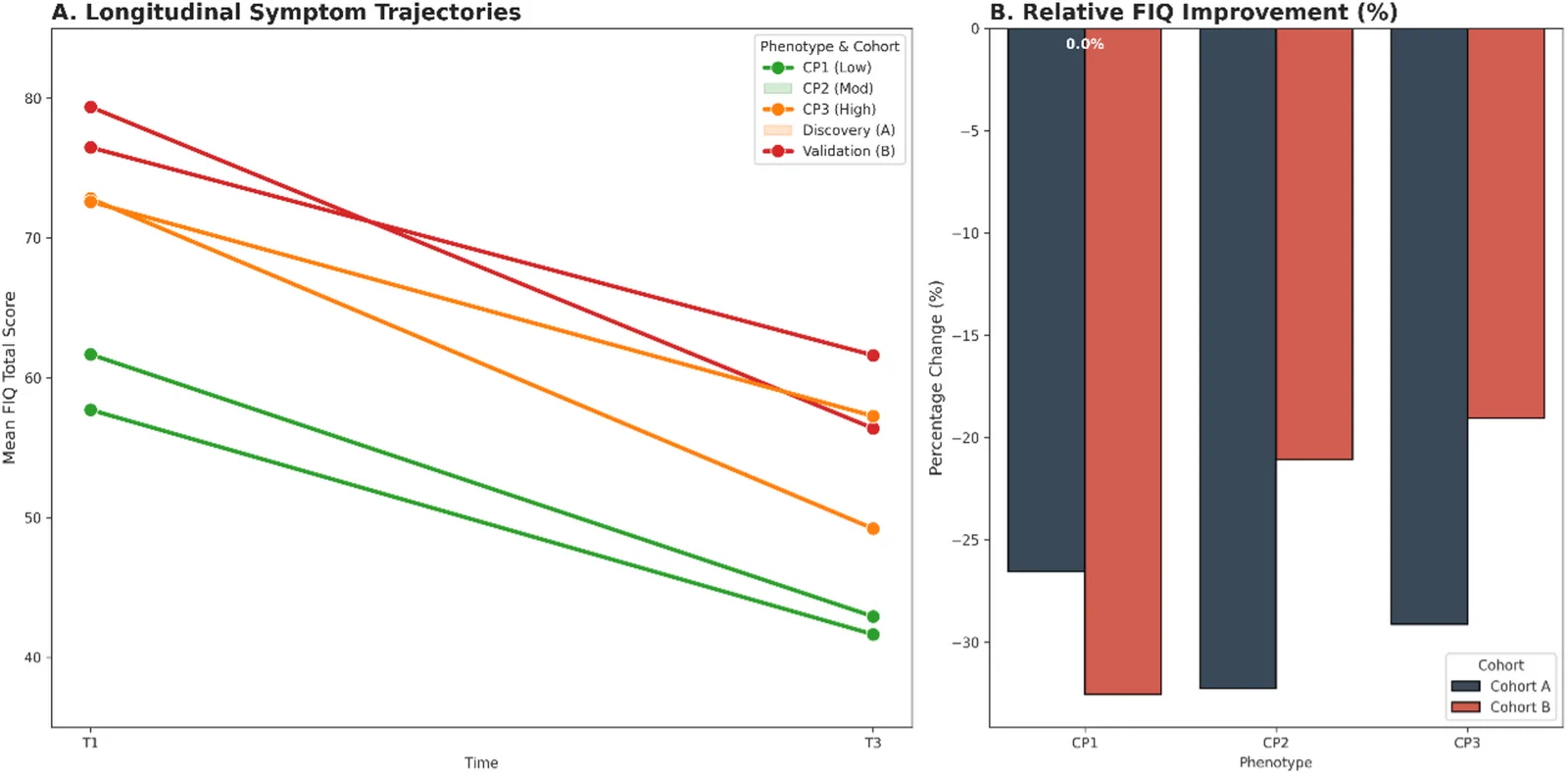
Cross-cohort validation of longitudinal symptom trajectories. **A**) Mean FIQ Total Score trajectories from baseline (T1) to final follow-up (T3) across symptom-based computational phenotypes in the discovery (Cohort A) and validation (Cohort B) cohorts. (**B**) Relative change in FIQ Total Score (%) by phenotype and cohort. Parallel trajectory patterns and consistent effect sizes across cohorts support the external validity of the computational phenotyping framework

Trajectory patterns were consistent between Cohort A and Cohort B (Fig. 7).

- Relative ordering of phenotypes was preserved.
- Parallel improvement trends were observed across datasets.

#### Effect size consistency

The magnitude of clinical improvement was reproducible across cohorts:

- CP2 showed the largest response in both datasets.
- CP1 showed moderate improvement.
- CP3 showed smaller but consistent changes.

#### Transition consistency

Transition patterns were comparable across cohorts (Table 6).

- CP1 remained highly stable (> 90%).
- CP2 consistently transitioned toward CP1.
- CP3 showed heterogeneous response patterns.

Although transition proportions differed slightly, the overall structure of phenotype evolution was preserved.

Transition patterns between baseline (T1) and final follow-up (T3) were compared across cohorts (Table 6).

**Table 6:** Cross-cohort comparison of longitudinal transition patterns between baseline (T1) and final follow-up (T3)

Baseline Phenotype	Follow-up Status	Cohort A (%)	Cohort B (%)	Difference (Δ)
CP1 (Low Impact)	Stable (Remains CP1)	92.75%	93.33%	+ 0.58%
	Worsening (to CP2/CP3)	7.25%	6.67%	−0.58%
CP2 (Moderate)	Transition to CP1	73.08%	56.25%	−16.83%
	Stable/Worsening	26.92%	43.75%	+ 16.83%
CP3 (High Impact)	Transition to CP1	56.84%	42.00%	−14.84%
	Transition to CP2	28.42%	28.00%	−0.42%
	Remained in CP3	14.74%	30.00%	+ 15.26%

Notes: Values represent the percentage of patients transitioning from each baseline phenotype to follow-up states between T1 (baseline) and T3 (final follow-up). Differences (Δ) indicate the absolute variation between the discovery cohort (A) and the validation cohort (B)

## Discussion

In this study, we present a reproducible computational framework for the identification and longitudinal validation of clinical symptom phenotypes in fibromyalgia using real-world registry data. By integrating a structured data curation pipeline, unsupervised learning, and cross-cohort validation strategies, our approach addresses key methodological challenges associated with heterogeneity and reproducibility in real-world evidence (RWE) settings.

It is important to distinguish the methodological robustness of the computational framework presented here from the conceptual scope of the findings. The clustering architecture, bootstrap stability, and frozen-model transferability demonstrate a resilient pipeline for phenotype discovery; however, the resulting phenotypes reflect the specific symptom dimensions captured by traditional diagnostic instruments (FIQ, WPI, SSS). Thus, while the framework provides a reproducible methodological platform, the phenotypic structure is bounded by the conceptual frameworks of the clinical instruments applied.

### Principal findings

The proposed framework identified three clinically interpretable symptom-based computational phenotypes that were structurally stable and reproducible across independent cohorts. These phenotypes—low impact (CP1), pain-dominant (CP2), and high multidimensional burden (CP3)—captured meaningful differences in baseline symptom profiles and exhibited distinct patterns of longitudinal evolution.

Importantly, the clustering structure demonstrated robustness through bootstrap stability analysis and maintained consistency when transferred to external datasets without model re-fitting. This finding supports the validity of a fixed-parameter phenotyping strategy in heterogeneous clinical environments.

Beyond cross-sectional differentiation, the identified phenotypes were associated with distinct longitudinal trajectories. While all groups exhibited clinical improvement, the magnitude and dynamics of response varied, with CP2 showing the largest relative improvement and CP3 demonstrating more heterogeneous outcomes. These patterns indicate that baseline phenotypic structure is informative of subsequent clinical evolution.

### Reproducibility and generalizability in real-world data

A central contribution of this work is the explicit focus on reproducibility across independent cohorts. Many existing phenotyping studies in fibromyalgia are limited to single-center analyses or lack external validation, reducing their generalizability.

In contrast, our framework enforces strict separation between model development and validation through a frozen clustering architecture. The preservation of phenotype structure across cohorts, as evidenced by consistent centroid profiles and comparable longitudinal trajectories, suggests that the identified phenotypes capture intrinsic clinical patterns rather than cohort-specific artefacts.

This approach aligns with current priorities in biomedical informatics, where reproducibility and transportability are critical for translating machine learning models into clinical practice.

### Interpretation of phenotype structure

The three identified phenotypes reflect distinct dimensions of symptom burden in fibromyalgia. CP1 represents a relatively homogeneous group with low severity and high longitudinal stability. CP3, in contrast, shows a compact but high-burden profile, with strong clustering stability but more variable clinical trajectories, suggesting differential treatment response within severe cases.

CP2 emerges as a particularly interesting subgroup characterized by disproportionately elevated pain relative to other symptom dimensions. Its higher mobility and greater improvement suggest that pain-dominant patients may respond differently to neuromodulatory interventions, highlighting the potential value of phenotype-guided therapeutic stratification.

The observed heterogeneity in stability and longitudinal behavior across phenotypes further supports the notion that fibromyalgia is not a uniform condition but comprises distinct clinical subpopulations.

### Methodological considerations

From a methodological perspective, this study emphasizes the importance of rigorous preprocessing, stability assessment, and validation in symptom-based computational phenotyping pipelines. The use of a structured data curation process was essential to mitigate noise and inconsistencies inherent to real-world registries.

The decision to restrict clustering to baseline variables ensured interpretability and prevented temporal data leakage, allowing longitudinal analyses to be conducted independently of phenotype definition. Additionally, the use of bootstrap-based stability metrics provided quantitative evidence that the identified clusters were not artifacts of sampling variability.

Crucially, the fixed-model transfer strategy—where scaling parameters and cluster centroids are applied unchanged to external datasets—offers a transparent and reproducible alternative to re-trained models. Beyond reproducibility, this architecture ensures high computational efficiency. By avoiding the need for resource-intensive model re-fitting, the framework reduces computational complexity to simple distance calculations relative to fixed centroids, enabling low-latency phenotype assignment. Given the low dimensionality of the feature space, this process can be executed in near real time using standard clinical hardware. As a result, the proposed framework is highly scalable and suitable for deployment in routine clinical workflows with minimal infrastructure requirements.

### Clinical implications in the context of fibromyalgia challenges

The identification of stable and transferable phenotypes has profound implications specifically tailored to the unique clinical challenges of fibromyalgia. As a complex disorder characterized by an incompletely understood etiology and a complete lack of definitive diagnostic biomarkers, fibromyalgia has historically been plagued by clinical scepticism, diagnostic delays, and persistent social stigma, where patients frequently face the distress of having their severe symptoms dismissed as psychosomatic or ‘all in their head.’ In this challenging landscape, our reproducible symptom-based computational phenotyping framework offers an objective, data-driven methodology to validate and map patient suffering. By showing that individual symptom profiles naturally cluster into highly stable, structured, and reproducible clinical symptom phenotypes (CP1–CP3) across independent cohorts, this framework helps reinforce diagnostic confidence for clinicians and provides objective validation of the heterogeneous ways the disease manifests.

Furthermore, this framework directly supports clinical stratification in a population where treatment response is notoriously unpredictable. Instead of relying on trial-and-error management, clinicians can utilize the ‘frozen model’ strategy to instantaneously map a patient’s baseline symptom vector to a defined phenotype. The distinct longitudinal trajectories observed—such as the high therapeutic responsiveness of the pain-dominant CP2 group to Li-rTMS compared to the more recalcitrant CP3 cohort—can guide clinicians in setting realistic prognostic expectations, optimizing healthcare resource utilization, and personalizing neuromodulatory or multidisciplinary intervention strategies. Ultimately, by providing a scalable, low-latency stratification tool, this framework can help transition fibromyalgia care from generic treatment paths to personalized, evidence-based management, directly mitigating the historical barriers to effective patient care.

### Limitations

This study has several limitations. First, the observational nature of the registry data limits causal inference regarding treatment effects. While longitudinal improvements were observed, these cannot be directly attributed to the intervention without controlled comparisons.

Second, the feature space was restricted to core clinical variables derived from established diagnostic instruments (FIQ, WPI, SSS). Although this enhances clinical interpretability and ensures consistency, these legacy instruments represent established diagnostic and functional constructs rather than the entirety of the patient experience. Crucially, unlike modern patient-reported outcome measures, these instruments were not developed using contemporary patient-centred qualitative methodologies that begin with direct, comprehensive symptom-elicitation and prioritization from patients themselves. Consequently, highly impactful symptom domains or life impacts that are meaningful to individuals living with fibromyalgia but are not represented within these traditional measures could not contribute to phenotype formation. Therefore, the identified phenotypes should be interpreted as groupings within the symptom dimensions emphasized by current diagnostic criteria, rather than comprehensive disease phenotypes. Future research should integrate broader, patient-centred outcome data to expand the feature matrix and explore whether latent patterns emerge beyond these established constructs (see Future Directions).

Third, cohort stratification was based on data availability rather than random assignment, which may introduce selection bias. However, this design reflects real-world clinical practice and strengthens ecological validity.

Finally, while external validation was performed across independent cohorts within the same registry framework, further validation in geographically and clinically diverse populations is needed.

### Boundary analysis and ambiguity

While the identified phenotypes demonstrate robust stability and clinical validity, certain “boundary cases”—patients exhibiting characteristics intermediate between CP1 and CP2 or CP2 and CP3—present higher classification uncertainty. These cases represent an inherent challenge in a continuous spectrum condition like fibromyalgia. Rather than true “failures” of the clustering architecture, these instances reflect the clinical reality of a gradient-based disease, where distinct labels are imposed upon a continuous symptomatic space.

Importantly, these boundary cases are visually reflected in the multidimensional heatmap (Fig. 4), where the intermediate phenotype (CP2) exhibits heterogeneous and partially overlapping feature patterns across clinical dimensions.

Future work should explore soft-clustering or probabilistic assignment methods to better characterize these high-uncertainty patients.

### Future directions

Future work should explore the integration of additional data modalities—including patient-centred qualitative outcomes, biomarkers, imaging, and digital health data—to refine phenotype definitions and move beyond the constraints of current diagnostic instruments. The extension of this framework to other chronic conditions may also provide insights into the generalizability of symptom-based computational phenotyping approaches.

Additionally, an essential next step will be to leverage the longitudinal phenotypes and transition probabilities identified by this framework to build predictive models for long-term clinically meaningful outcomes. Specifically, future research should evaluate whether baseline phenotype assignment can prospectively predict individual symptom progression, long-term treatment response stability, healthcare utilization rates, and the likelihood of transition between phenotypic subgroups over time. Developing these predictive capabilities will be crucial to transition these symptom-based computational phenotypes into actionable, personalized clinical decision-support systems that directly improve clinical workflow efficiency.

Moreover, prospective studies evaluating the clinical utility of phenotype-guided interventions are essential. By applying this framework to broader patient-derived datasets, future work will be better positioned to translate these clinical symptom phenotypes into actionable, personalized treatment strategies that directly improve patient outcomes.

### Framework adaptability and future applications

A key strength of the presented methodology is its independence from specific feature sets. While this study utilized standard diagnostic instruments to ensure immediate applicability across real-world registries, the proposed clustering architecture is inherently modular and scalable. The framework is highly adaptable to richer patient-derived datasets, including variables identified through qualitative patient-centred methodologies. Future efforts to populate this framework with broader symptom domains—capturing the full spectrum of disease impact—are expected to reveal more nuanced phenotypic structures, demonstrating the utility of this approach as a flexible foundation for personalized fibromyalgia research.

## Conclusions

This study presents a reproducible symptom-based computational phenotyping framework for the identification and longitudinal validation of clinical subgroups in fibromyalgia using real-world registry data. By integrating structured data curation, unsupervised learning, and cross-cohort validation under a fixed-model architecture, the proposed approach enables the extraction of stable and transferable clinical symptom phenotypes.

The identified phenotypes demonstrated consistent multidimensional structure and reproducible longitudinal trajectories across independent cohorts, supporting their robustness in heterogeneous real-world settings. These findings highlight the value of combining baseline phenotyping with longitudinal analysis to capture both static and dynamic aspects of complex chronic conditions.

The framework provides a scalable and generalizable approach for symptom-based computational phenotyping in biomedical informatics and may support future applications in patient stratification, outcome prediction, and personalized treatment strategies in fibromyalgia and other chronic diseases.

## Supplementary Information

Below is the link to the electronic supplementary material.

Supplementary Material 1

Supplementary Material 2

## Data Availability

The reproducible computational framework developed in this study, including the Python code for the data curation pipeline, clustering analysis, and longitudinal visualization, is openly available in the GitHub repository: https://github.com/nazariofelix-CTB/fibromyalgia-rTMS-trajectories. This repository also contains the technical outputs supporting the results, such as cluster summary tables, transition counts, and validation metrics. The minimal dataset necessary to replicate the findings, fully anonymized in compliance with data protection regulations, is available in the Zenodo repository, https://doi.org/10.5281/zenodo.20272363 [12]. Additional raw clinical documentation and datasets that support the findings of this study are available from the corresponding author on reasonable request, but restrictions apply to the availability of these data due to the privacy policies of the participating clinical centers and are not publicly available.

## Abbreviations

CP: Symptom-Based Computational Phenotype
CP1: Symptom-Based Computational Phenotype 1 (Low Symptom Burden)
CP2: Symptom-Based Computational Phenotype 2 (Pain-Dominant)
CP3: Symptom-Based Computational Phenotype 3 (High Multidimensional Burden)
FAIR: Findable, Accessible, Interoperable, and Reusable
FIQ: Fibromyalgia Impact Questionnaire
Li-rTMS: Low-intensity repetitive Transcranial Magnetic Stimulation
MDR: Medical Device Regulation
PROs: Patient-Reported Outcomes
RWD: Real-World Data
RWE: Real-World Evidence
SSS: Symptom Severity Scale
WPI: Widespread Pain Index
