# Supplementary material for: A reproducible symptom-based computational phenotyping framework for longitudinal fibromyalgia analysis using real-world registry data: a cross-cohort validation study
Source: BMC Med Inform Decis Mak. 2026 Aug 5;26:446. doi: 10.1186/s12911-026-03740-6 (PMC13726113; doi:10.1186/s12911-026-03740-6)
Supplement: Supplementary file 1 [file 12911_2026_3740_MOESM1_ESM.docx]

Supplementary Figure SF 1.Sensitivity analysis of the clustering structure: comparison between K-means++ and K-medoids (Gower’s distance).

The heatmap illustrates the cross-tabulation of cluster assignments between the original K-means++ model (rows) and the K-medoids approach (columns). Each cell represents the number of patients assigned to each computational phenotype (CP1–CP3) by both methods. The comparison reveals that agreement is higher for the extreme phenotypes (CP1 and CP3), while the intermediate phenotype (CP2) shows substantial dispersion across adjacent clusters. This pattern indicates that boundary cases are sensitive to the choice of distance metric. The Adjusted Rand Index (ARI = 0.196) reflects limited agreement at the individual assignment level. However, despite these differences, the hierarchical structure of symptom severity is preserved across both approaches, with consistent ordering of phenotypes (CP1 < CP2 < CP3). These findings support the interpretation of fibromyalgia as a continuous clinical spectrum rather than a set of strictly separable clusters.
