# Supplementary material for: A reproducible symptom-based computational phenotyping framework for longitudinal fibromyalgia analysis using real-world registry data: a cross-cohort validation study
Source: BMC Med Inform Decis Mak. 2026 Aug 5;26:446. doi: 10.1186/s12911-026-03740-6 (PMC13726113; doi:10.1186/s12911-026-03740-6)
Supplement: Supplementary file 2 [file 12911_2026_3740_MOESM2_ESM.docx]

Supplementary Table ST 1: Temporal adherence and longitudinal follow-up intervals across clinical sites in the Discovery Cohort (n=268).

| Clinical Centre | Patients (n) | T1-T2 (weeks) | T2-T3 (weeks) | T1-T3 (weeks) |
| --- | --- | --- | --- | --- |
| Centre 1 | 149 | 2.91 ± 0.19 | 2.99 ± 7.61 | 5.96 ± 7.58 |
| Centre 2 | 109 | 4.31 ± 4.80 | 7.26 ± 9.23 | 11.61 ± 9.64 |
| Others | 10 | 6.84 ± 11.90 | 4.13 ± 4.21 | 10.97 ± 12.35 |

Notes: Data are presented as mean ± standard deviation (SD). Temporal intervals refer to the duration between baseline (T1), intermediate (T2), and final (T3) assessments. To maintain institutional anonymity, clinical sites are categorized as Centre 1 and Centre 2 (high-volume centers) and 'Others' (grouping peripheral sites with n < 5). Statistical comparison between the two high-volume centers (Centre 1 vs. Centre 2) using Welch’s t-test yielded significant differences (p < 0.01 for all intervals). However, these fluctuations reflect real-world clinical practice and patient-specific scheduling rather than protocol deviations. Sensitivity analysis confirmed that these temporal variations did not act as confounding variables for the phenotypic transition probabilities reported in the main study.
